# Kinetic Pattern Recognition in Home-Based Knee Rehabilitation Using Machine Learning Clustering Methods on the Slider Digital Physiotherapy Device: Prospective Observational Study

**DOI:** 10.2196/69150

**Published:** 2025-03-18

**Authors:** Clement Twumasi, Mikail Aktas, Nicholas Santoni

**Affiliations:** 1 Nuffield Department of Medicine Experimental Medicine Division University of Oxford Oxford United Kingdom; 2 Department of Bioengineering Imperial College London South Kensington, London United Kingdom; 3 Department of Biology University of North Carolina Greensboro, NC United States

**Keywords:** machine learning, cluster analysis, force measurement, knee replacement, musculoskeletal, physical therapy, Slider device, knee osteoarthritis, digital health, telerehabilitation

## Abstract

**Background:**

Recent advancements in rehabilitation sciences have progressively used computational techniques to improve diagnostic and treatment approaches. However, the analysis of high-dimensional, time-dependent data continues to pose a significant problem. Prior research has used clustering techniques on rehabilitation data to identify movement patterns and forecast recovery outcomes. Nonetheless, these initiatives have not yet used force or motion datasets obtained outside a clinical setting, thereby limiting the capacity for therapeutic decisions. Biomechanical data analysis has demonstrated considerable potential in bridging these gaps and improving clinical decision-making in rehabilitation settings.

**Objective:**

This study presents a comprehensive clustering analysis of multidimensional movement datasets captured using a novel home exercise device, the “Slider”. The aim is to identify clinically relevant movement patterns and provide answers to open research questions for the first time to inform personalized rehabilitation protocols, predict individual recovery trajectories, and assess the risks of potential postoperative complications.

**Methods:**

High-dimensional, time-dependent, bilateral knee kinetic datasets were independently analyzed from 32 participants using four unsupervised clustering techniques: k-means, hierarchical clustering, partition around medoids, and CLARA (Clustering Large Applications). The data comprised force, laser-measured distance, and optical tracker coordinates from lower limb activities. The optimal clusters identified through the unsupervised clustering methods were further evaluated and compared using silhouette analysis to quantify their performance. Key determinants of cluster membership were assessed, including demographic factors (eg, gender, BMI, and age) and pain levels, by using a logistic regression model with analysis of covariance adjustment.

**Results:**

Three distinct, time-varying movement patterns or clusters were identified for each knee. Hierarchical clustering performed best for the right knee datasets (with an average silhouette score of 0.637), while CLARA was the most effective for the left knee datasets (with an average silhouette score of 0.598). Key predictors of the movement cluster membership were discovered for both knees. BMI was the most influential determinant of cluster membership for the right knee, where higher BMI decreased the odds of cluster-2 membership (odds ratio [OR] 0.95, 95% CI 0.94-0.96; *P*<.001) but increased the odds for cluster-3 assignment relative to cluster 1 (OR 1.05, 95% CI 1.03-1.06; *P*<.001). For the left knee, all predictors of cluster-2 membership were significant (.001≤*P*≤.008), whereas only BMI (*P*=.81) could not predict the likelihood of an individual belonging to cluster 3 compared to cluster 1. Gender was the strongest determinant for the left knee, with male participants significantly likely to belong to cluster 3 (OR 3.52, 95% CI 2.91-4.27; *P*<.001).

**Conclusions:**

These kinetic patterns offer significant insights for creating personalized rehabilitation procedures, potentially improving patient outcomes. These findings underscore the efficacy of unsupervised clustering techniques in the analysis of biomechanical data for clinical rehabilitation applications.

## Introduction

### Background of the Study

The challenge of optimizing knee rehabilitation exercises following arthroplasty or injury is critical, given the high prevalence of knee surgeries and the need for effective recovery strategies. According to the *American Joint Replacement Registry 2022 Annual Report*, over 2.5 million knee and hip arthroplasty cases were documented, constituting a significant proportion of knee replacements. The quality and effectiveness of the ensuing rehabilitation, which has historically relied on patient self-reporting, subjective assessments, and poor compliance, is crucial to the success of these procedures. Such methods can lead to suboptimal outcomes, as they fail to capture biomechanical data that could inform treatment planning and postoperative complications [1,2].

Recent developments in sensor technology have made it possible to collect detailed kinetic data during physical therapy exercises. This presents a chance to transition from subjective assessments to more objective, data-driven methods. This change is especially significant in home-based rehabilitation, where it is crucial to collect valuable data consistently. The newly developed Slider device [3] exemplifies this technological advancement by capturing multidimensional movement data, including force and spatial coordinates, directly from patients’ lower limbs during exercise. The Slider device is the first rehabilitation device capable of remotely collecting force and motion data in a patient’s home. This capability enhances the ability to monitor patients in real time and to produce more informative datasets that capture the dynamic interaction of force and movement in a real-world, home-based setting [4].

A recent study has extensively examined the efficacy of the Slider device for prehabilitation prior to total knee replacement surgery [3]. The study revealed that the device can assist patients in performing autonomous physiotherapy prior to surgery in nonclinical environments. The research involved 17 patients awaiting knee replacement surgery at a UK National Health Service hospital. Their findings confirmed the device’s practicality and user-friendliness for patient-initiated exercises, with no recorded safety issues or adverse occurrences [3]. Principal findings revealed improvements in user involvement and physical preparedness. The study advised that subsequent research should involve a bigger and more diverse participant cohort to comprehensively validate its findings regarding the device. In order to enhance our understanding of this new device, additional studies may be required to investigate the multidimensional kinetic and kinematic data from the Slider using unsupervised clustering methods for the first time across a comparatively larger and diverse population. This exploration is necessary to identify potential movement patterns that could offer further insights into workout performance and recovery. The insights gained from these computational methods may improve comprehension of patient heterogeneity, enabling customized rehabilitation programs that more effectively address individual needs and improve outcomes for diverse groups.

The user datasets from this new data-collection device represent a significant contribution to the field, as they provide a new level of detail in understanding how kinetic patterns evolve during rehabilitation outside of a clinical environment. Despite these advancements, analyzing such high-dimensional, time-dependent data remains a complex challenge. Previous studies have applied clustering methods to other rehabilitation data to identify movement patterns and predict recovery outcomes. However, these efforts have yet to use force or motion data captured outside of a clinical environment, limiting their ability to inform clinical decisions [5,6].

Data were collected from healthy volunteers performing lying flexions and extensions of their knees, including relevant demographic characteristics and pain levels. K-means, hierarchical clustering, partition around medoids (PAM), and CLARA (Clustering Large Applications) were applied to identify distinct movement patterns (based on force and displacement measurements as primary outcomes). Performance evaluations were conducted using the classical silhouette analysis to determine the most appropriate clustering method. Then, post hoc analyses were performed with multinomial logistic regression to explore potential demographic predictors of cluster membership based on the best models for the right and left knee datasets, respectively. This study contributes to the understanding of rehabilitation dynamics beyond the clinical setting. The insights gained from the uniquely identified data clusters may therefore enable clinicians to make more informed, data-driven decisions, ultimately enhancing the precision and effectiveness of rehabilitation programs. The primary aim of this study is to advance the understanding of kinetic patterns during knee rehabilitation by applying and comparing four unsupervised clustering algorithms to new datasets collected for the first time using the Slider device.

### Paper Structure and Research Questions

This study is structured into five main sections. The *Introduction* section provides an overview of the study’s background, outlines the paper’s organization, and highlights its primary contributions. The *Methods* section outlines the empirical kinetic data used in the study, participant recruitment, study design, and ethical considerations, followed by a concise summary of the clustering methodologies and additional modeling considerations. The *Results* section presents the findings derived from the statistical analysis of the experimental data. Finally, the final section (*Discussion*) offers a discussion of the key findings, draws conclusions, and suggests directions for future research. With the help of experimental bilateral knee kinetic datasets obtained from the newly developed home-based rehabilitation device (Slider), this study seeks to address the following open research questions:

- What distinct movement patterns can be identified from multidimensional kinetic data during home physiotherapy?
- Can real-time clustering of movement data provide meaningful feedback for personalized physiotherapy?
- How effective is cluster analysis in distinguishing between varying levels of movement efficiency and recovery states?
- How do kinetic movement patterns evolve over the course of physiotherapy, as detected through the new Slider device?
- Can the multidimensional movement data collected remotely provide reliable insights for monitoring physiotherapy outcomes?

## Methods

### Data Collection, Recruitment, and Study Design

Continuously observed, time-dependent, and high-dimensional datasets were collected from both knees of 32 participants (totaling 64 time-varying datasets with large data points) using Slider during lying flexion and extension exercises. Participants were not required to have undergone any prior treatment. Demographic characteristics such as gender (21 male and 11 female participants), ethnicity (30 White participants and 2 mixed White and Asian participants), laterality (29 right and 3 left), age (mean 47.3, SD 2.11; range 21-77 years), height (cm), and weight (kg) were recorded. Each participant’s BMI was calculated from their height and weight measurements (mean 26.2, SD 4.99; range 18.3-40.6 kg/m^2^). Pain levels or scores were quantified using a standard analog pain scale, ranging from a score of 0=no pain to 10=worst imaginable pain.

**Figure 1:**
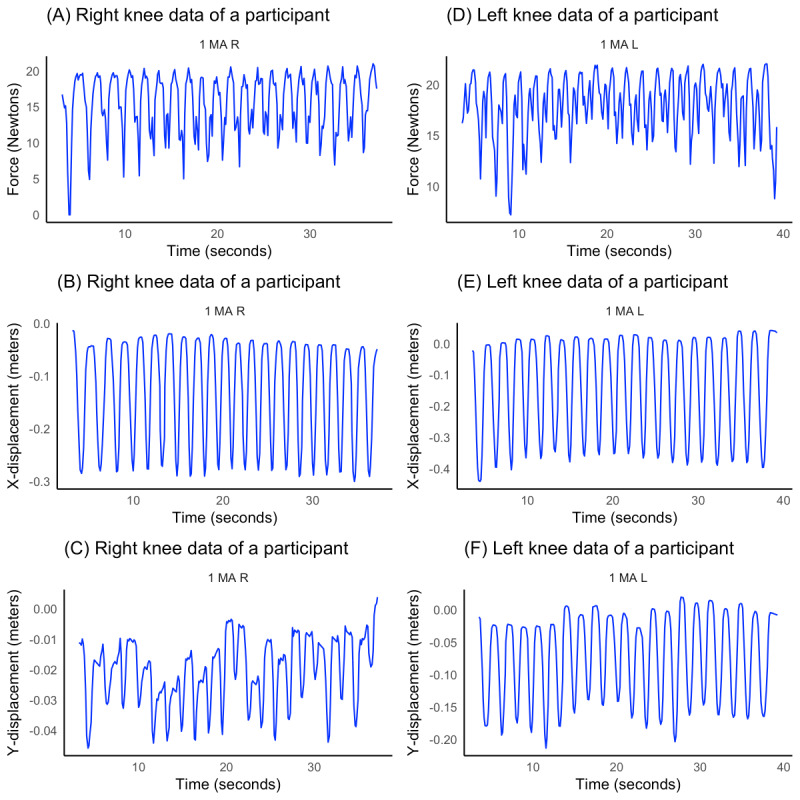
Time-series plots illustrating the unadjusted observed kinetic data, including force and X-Y displacements, recorded at the (A-C) right and (D-F) left knees of a study participant (ID: 1 MA). These plots provide an overview of the raw movement patterns captured by the Slider device, highlighting temporal variations in force exertion and spatial displacement during rehabilitation exercises.

Participants were not excluded from the study due to deformities or complications from prior surgeries or therapy treatments. The study was conducted without supervision, as it was intended to be performed remotely in the participants’ homes due to the inherently mobile nature of the Slider device. Consequently, the study was deemed low risk and noninvasive. Each participant completed 20 cycles, at a self-selected speed with the device recording force (in Newtons) and X-Y displacement (in meters) measured with an optical tracker. The data output was time stamped (in seconds). Figure 1 is a time-series visualization of observed data of the main unadjusted outcome variables for clustering of a study participant. All statistical tests, with a significance level set at *P*<.05, and cluster analyses were performed in R statistical software (version 4.3.1; R Core Team) [7].

The participants comprised the entire existing pool of volunteers registered with the Slider device manufacturer. These individuals were invited to participate based on their prior engagement with the device, ensuring familiarity with its use. Recruitment procedures followed ethical guidelines, with all participants providing informed consent before data collection. This study follows a machine learning (ML) predictive modeling design in biomedical research, using unsupervised clustering techniques to analyze high-dimensional kinetic data and identify distinct movement patterns in home-based knee rehabilitation.

### Ethical Considerations

This low-risk and noninvasive study was classified as a service evaluation in accordance with the guidelines from the UK Health Research Authority. Consultation with the UK Research and Innovation Medical Research Council using Integrated Research Application System (348336) confirmed that the study adhered to all relevant ethical standards, and therefore, formal ethical committee approval was not required. All participants provided informed consent before participating, with explicit consent obtained for both data collection and subsequent analysis. To protect privacy and confidentiality, all study data were fully anonymized before analysis, ensuring no personally identifiable information was included. No financial compensation or other incentives were provided for participation, as the study was conducted on a voluntary basis. Finally, no identifiable images or personal data from participants are included in the study dataset or any supplementary materials.

### Data Preprocessing

Data preprocessing is a crucial step in medical data analysis, especially for high-dimensional datasets with missing or incomplete information. It ensures data integrity, accuracy, and usability by handling missing values (where applicable), normalizing distributions, and reducing dimensionality, ultimately improving predictive models and clustering techniques performance [8]. Consequently, a two-stage data preprocessing method was used on the time-dependent datasets before conducting cluster analysis. In the first stage, potential outliers in the time-series data for each participant were automatically detected and corrected using the *tsoutliers* R package [9], based on the force and X-Y displacement data. In the second stage, the cleaned time-series data underwent decomposition using locally estimated scatterplot smoothing (decomposition). This process aided in detrending the series for each participant, making them more stationary, and removing cyclical patterns to mitigate cyclical effects, thereby enhancing the accuracy of the clustering analysis results. Because outlier correction and locally estimated scatterplot smoothing decomposition effectively standardize the time-series data, decreasing noise and maintaining comparability between participants by addressing both extreme values and nonstationary trends, this two-stage approach is sufficient without additional data normalization. Reliable clustering outcomes are therefore possible since any residual variation in the data represents significant variations in participant movement patterns rather than artifacts from data scale or trend discrepancies.

### Unsupervised Clustering Methods

#### Overview

In this study, the conventional hierarchical clustering and three widely used partitional clustering algorithms, namely: k-means [10,11], PAM [10,12], and CLARA [10], were used and compared to respectively divide the preprocessed bilateral knee datasets into an optimal number of clusters (represented by *k*>1). This partitioning ensures that data points representing kinetic features within the same cluster exhibit greater similarity to each other than to those in other clusters. These clustering methods have their respective algorithmic processes for distinctly grouping data, each with advantages and disadvantages regarding its robustness, scalability, and applicability related to the data structure, dataset size, and noise sensitivity. These justify the choice of models for this study and highlight the need to identify the best clustering algorithms for the right and left knee kinetic datasets, which were continuously observed over time for each volunteer. A summary of the underlying clustering methods used in this study is presented below.

#### K-Means Clustering

K-means is a centroid-based clustering algorithm that partitions a set of *n* data points {*x*_1_, *x*_2_, ..., *x_n_*} ∈ R*^d^* into *k* clusters {*C*_1_, *C*_2_, ..., *C_k_*} by minimizing the within-cluster sum of squares objective function [10]:



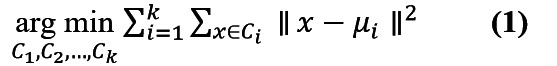



where μ*_i_* is the mean of the points in cluster *C_i_*. The algorithm starts with *k* initial centroids, assigns each point to the nearest centroid or cluster center, and updates centroids by computing the mean of assigned points. This process repeats until convergence, typically when cluster assignments no longer change. K-means is computationally efficient, with a time complexity of *O*(*n* ⋅ *k* ⋅ *d* ⋅ *t*), where *t* is the number of iterations; but is sensitive to the initial choice of centroids.

#### Hierarchical Clustering

Hierarchical clustering constructs a nested tree of clusters by either agglomerative (bottom-up) or divisive (top-down) methods. The agglomerative approach starts with each data point as a single cluster and iteratively merges the two closest clusters based on a chosen linkage criterion. For example, in single linkage, the distance between clusters *A* and *B* is defined as [13]:



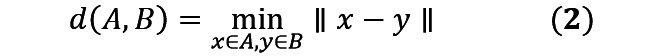



The agglomerative algorithm first computes the distance matrix for all pairs of points, then merges the closest clusters based on the chosen linkage criterion, and finally, updates the distance matrix, repeating this process until all points form a single cluster. The divisive algorithm starts with all data points in a single cluster and recursively splits them into smaller clusters until each data point forms its own cluster. At each step, it maximizes a splitting criterion such as the between-cluster variance denoted by:



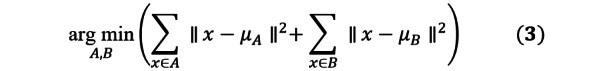



where *A* and *B* are the resulting clusters from the split, and their respective centroids are denoted by *μ_A_* and *μ_B_*. Hierarchical clustering is effective for detecting nested structures, but it has a time complexity of either *O*(*n*^2^log*_n_*) for the agglomerative method (making it effective for small to medium-sized datasets) or *O*(2*^n^*) for the divisive approach (making it less effective for large datasets).

#### PAM

PAM is a medoid-based clustering algorithm that extends k-means by selecting actual data points (medoids) as cluster centers, minimizing the sum of dissimilarities between points and their nearest medoid. The objective is to find a set of *k* medoids {*m*_1_, *m*_2_, ..., *m_k_*} that minimizes [10]:



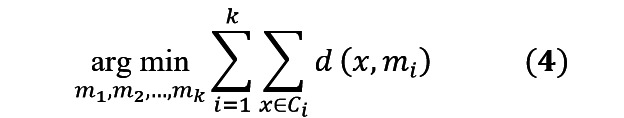



where *d*(*x*, *m_i_*) is the dissimilarity (eg, Euclidean distance) between a data point *x* and the medoid *m_i_* of cluster *C_i_*. PAM is robust to noise and outliers but has a higher computational cost of *O*(*k*[*n* – *k*]^2^), which can limit its scalability for large datasets. Different variants and extensions of PAM also exist [10].

#### CLARA

CLARA is an extension of PAM designed for large datasets. It draws multiple samples of the data and applies PAM to each sample, selecting the set of medoids that minimizes the clustering cost over the entire dataset [12]. CLARA reduces the computational burden of PAM by using random sampling, maintaining a complexity of *O*(*k*|*S*|^2^ + *k*[*n* – |*S*|]), where |*S*| is the sample size. CLARA scales better to large datasets, but its performance depends on the quality and representativeness of the random samples [10].

### Other Statistical Modeling Considerations and Performance Evaluation

To respectively determine the optimal number of unique clusters in the data for both knees, the *NbClust* R Package [14] was used. This package evaluates over 30 different cluster identification schemes or indices, with the optimal cluster size determined automatically using a majority rule. After clustering, the performance of the four ML approaches for the right and left knee datasets was evaluated and compared using silhouette analyses, which validate clustering methods [15]. Silhouette coefficients or scores, which range from –1 (indicating incorrect clustering) to +1 (indicating highly dense or correct clustering), were calculated for each cluster identified across the four data partitioning methods. Based on their distributions, appropriate pooled estimates of these scores were used to compare the models’ performance for the right and left knee data clustering, respectively. Principal component analysis (PCA) was adopted to visualize the clustered high-dimensional data {*x*_1_, *x*_2_, ..., *x_n_*} ∈ R*^d^* in a reduced dimensional space.

While the primary cluster analysis using force and displacement outcome data can potentially categorize movement patterns into distinct groups in the right and left knees, it remains essential to identify additional demographic factors that may influence these patterns and their clinical relevance [16]. Thus, a multinomial logistic regression model with analysis of covariance adjustment was used to investigate whether demographic variables such as age, gender, BMI, and pain scores could serve as significant predictors of cluster membership. This approach allows for a more comprehensive understanding of the determinants of cluster assignment by examining how these demographic factors, when adjusted for kinetic measurements, contribute to the likelihood of a participant belonging to a specific cluster at a particular time (since cluster membership is time-varying given the time-dependent kinetic data structure). Incorporating these variables enhances the ability to tailor personalized rehabilitation protocols by considering both biomechanical and individual patient characteristics, ultimately supporting more precise and effective clinical decision-making.

Using an analysis of covariance–based logistic model with adjustment for the actual observed force and X-Y displacement measurements, as well as temporal effects, the model is mathematically defined as follows:



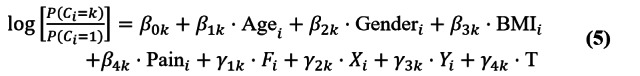



for each cluster category 1<*k*≤*K* relative to a baseline category (cluster 1), where *K* denotes the total number of uniquely identified clusters. *P*(*C_i_*=*k*) represents the probability of the *i*th individual being in cluster *k* at time *T*≥0, and the regression coefficients: *β*_1_*_k_*, *β*_2_*_k_*, *β*_3_*_k_*, and *β*_4_*_k_* represent the respective effects of the main demographic predictors (age, gender, BMI, and pain score) on the log-odds of being in cluster *k*≥2 versus the baseline cluster 1, adjusted for force (*F*), X-Y displacements (denoted by *X* and *Y*) and time of measurement (*T*).

The coefficients *γ*_1_*_k_*, *γ*_2_*_k_*, *γ*_3_*_k_*, and *γ*_4_*_k_* control for the influence of force, X-Y displacements, and time of measurement, respectively. The model was fitted with the help of the *nnet* R package [17]. Finally, the odds ratios (ORs) were estimated, which quantify the magnitude of the discriminatory effect of each of the main demographic predictors by exponentiating the estimated log-odds regression coefficients from exp(*β_gk_*) for 1≤*g*≤4 and 1<*k*≤*K*. The right and left knee datasets were respectively split into 80% training sets (for model fitting) and 20% testing sets for cross-validation to estimate the prediction accuracy from the resulting confusion matrix and the area under the receiver operating characteristic curve statistic.

## Results

### Cluster Analysis at Both Knees

#### Summary of Key Findings

**Figure 2:**
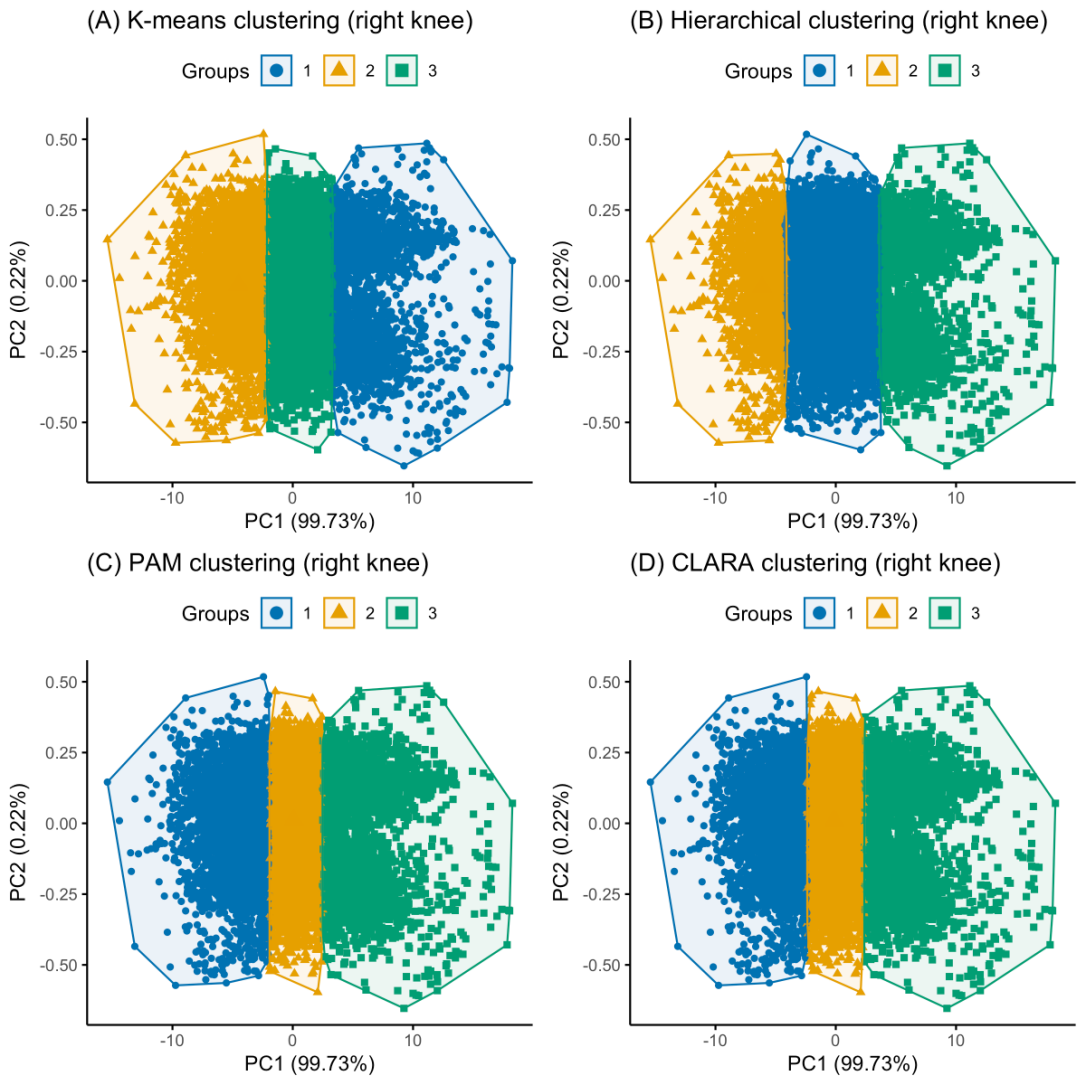
Principal component analysis visualizations comparing the three identified movement clusters at the right knee across the four clustering methods: (A) k-means, (B) hierarchical, (C) PAM, and (D) CLARA. These visualizations are based on adjusted kinetic features (force and X-Y displacements) and illustrate how different clustering techniques distinguish movement patterns in the multidimensional dataset. CLARA: Clustering Large Applications; PAM: partition around medoids.

**Figure 3:**
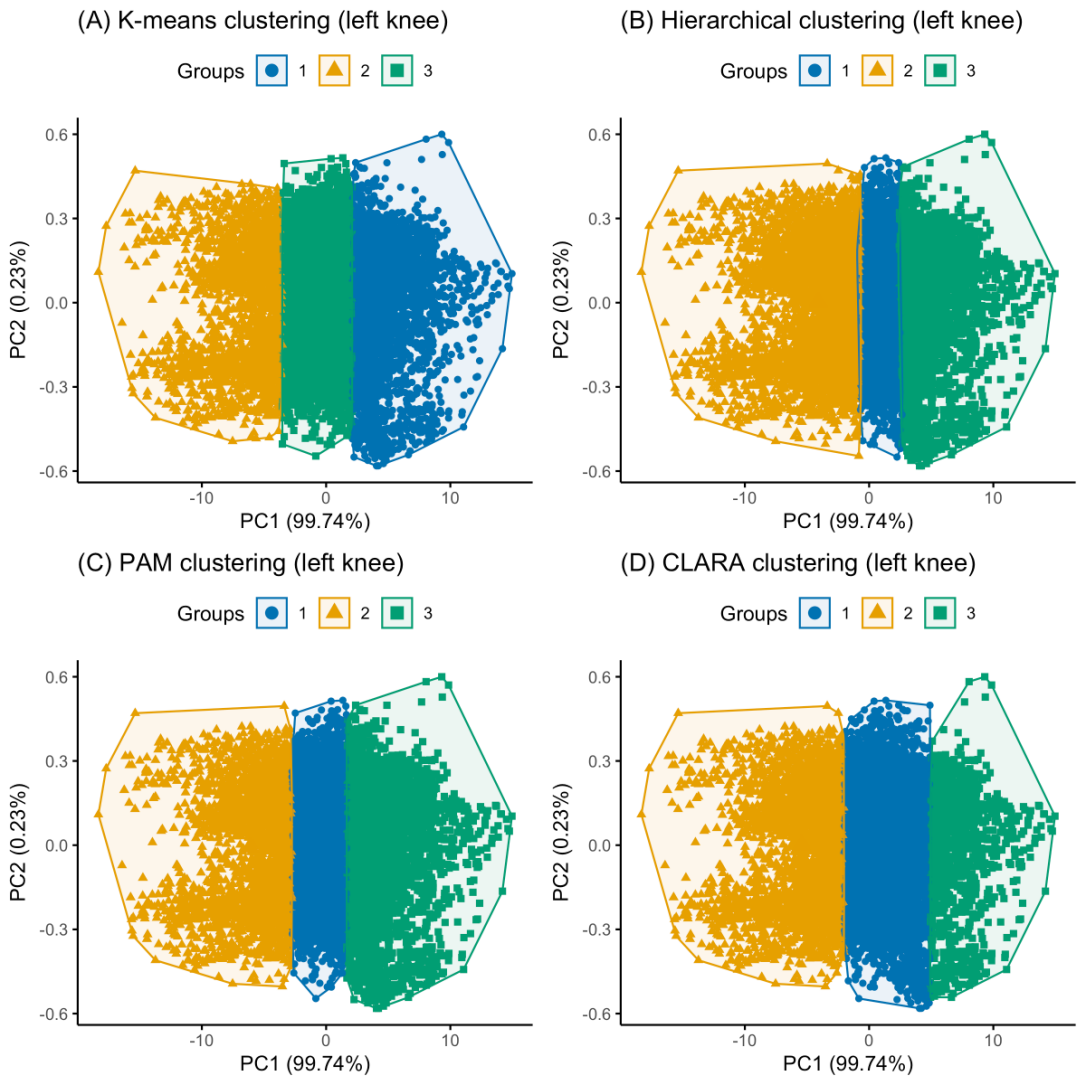
Principal component analysis visualizations comparing the three identified movement clusters at the left knee across the four clustering methods: (A) k-means, (B) hierarchical, (C) PAM, and (D) CLARA. These plots provide insights into the separability of kinetic movement patterns based on adjusted force and spatial displacement data. CLARA: Clustering Large Applications; PAM: partition around medoids.

**Figure 4:**
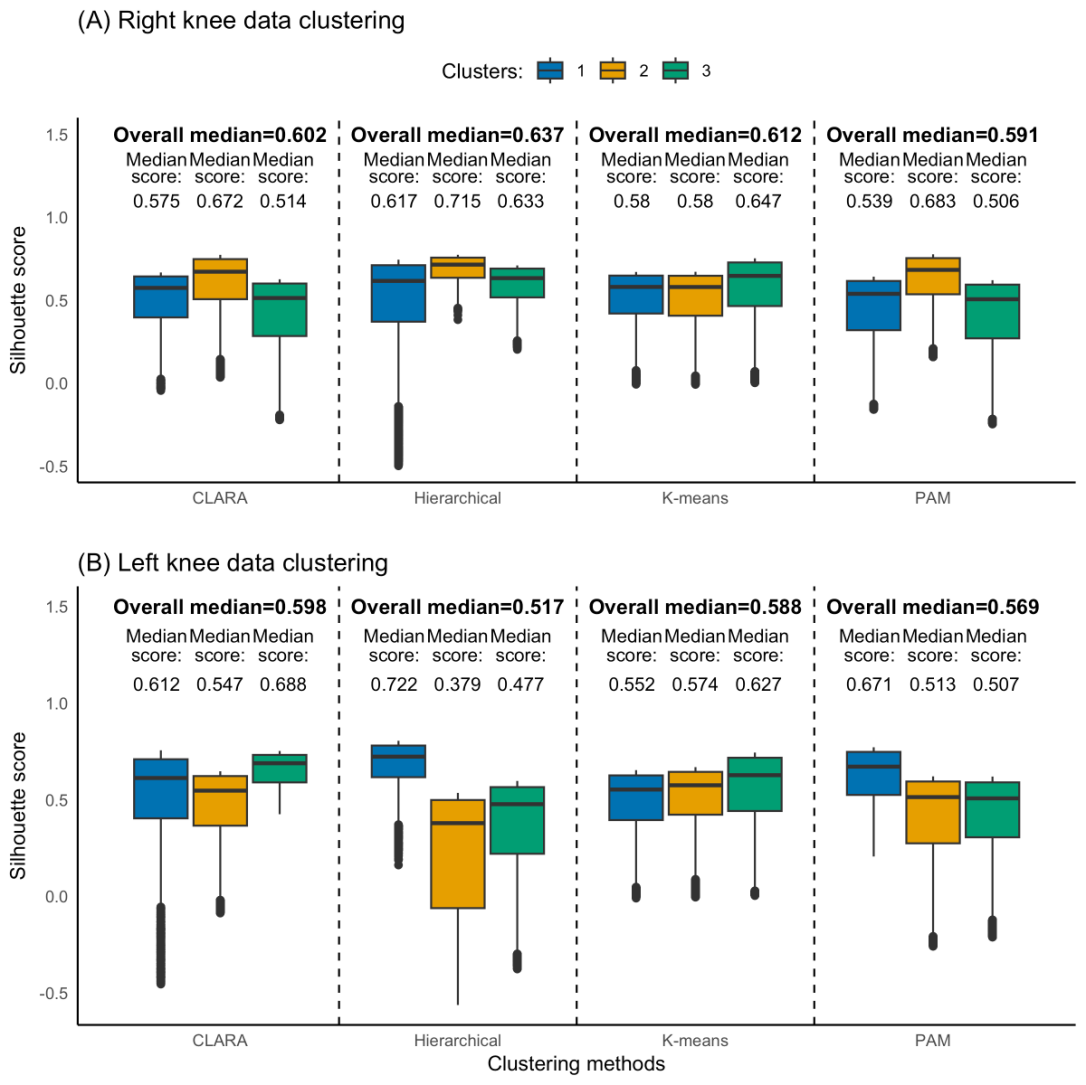
Distributional plot comparing estimated silhouette coefficients across the four clustering methods (k-means, hierarchical, PAM, and CLARA) at the (A) right and (B) left knees. The plot presents both cluster-specific and pooled estimates, highlighting the relative performance of each clustering method in distinguishing movement patterns from kinetic data at both knees. CLARA: Clustering Large Applications; PAM: partition around medoids.

**Figure 5:**
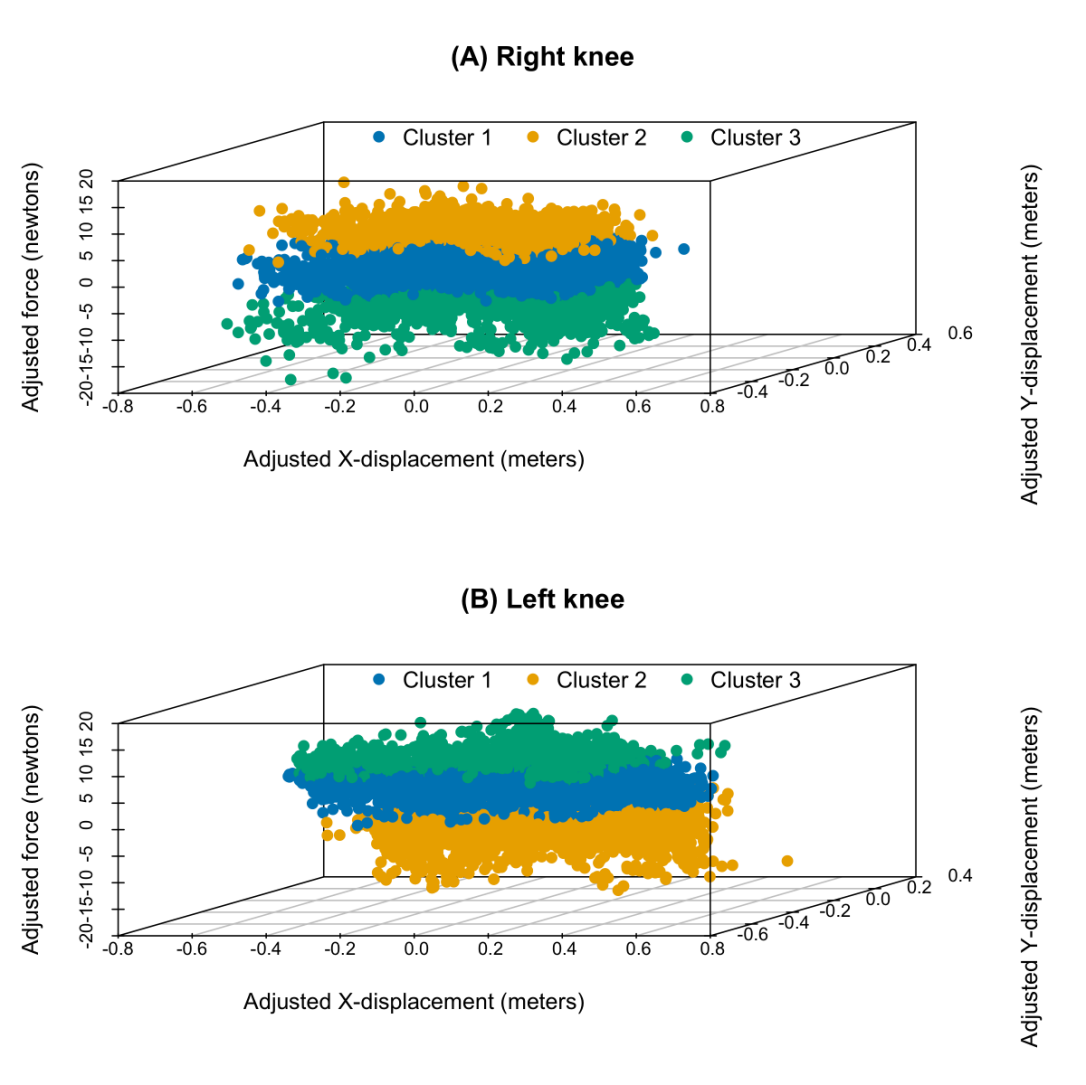
3D scatterplot depicting the clustering of adjusted force measurements (in Newtons) against adjusted X-Y displacements (in meters) for the (A) right and (B) left knees. The clustering is based on the most optimal knee-specific methods identified through silhouette analysis—hierarchical clustering for the right knee and CLARA for the left knee—demonstrating the spatial distribution of movement patterns within the dataset. CLARA: Clustering Large Applications.

Three clusters were optimally identified for both knees, each representing a unique kinetic pattern. K-means, hierarchical, PAM, and CLARA clustering were subsequently performed based on these predetermined clusters. Figures 2 and 3 show a PCA visualization of the clustered datasets in a lower-dimensional space for both knees. The silhouette analysis (Figure 4) revealed that hierarchical clustering was relatively found to be the most effective for the right knee kinetic datasets (with an average silhouette score of 0.637) followed by k-means (with an average silhouette score of 0.612), while CLARA proved to be the best-performing method for the left knee datasets (with an average silhouette score of 0.598) followed by k-means (with an average silhouette score of 0.588). It can be inferred from these results that the right knee datasets resulted in a better clustering result than the left knee datasets. Figure 5 presents a scatterplot of the adjusted kinetic features (which have been detrended and cycle-adjusted) in a 3D space for the right and left knees, stratified by the identified clusters, based on the best-performing, knee-specific clustering algorithms.

**Figure 6:**
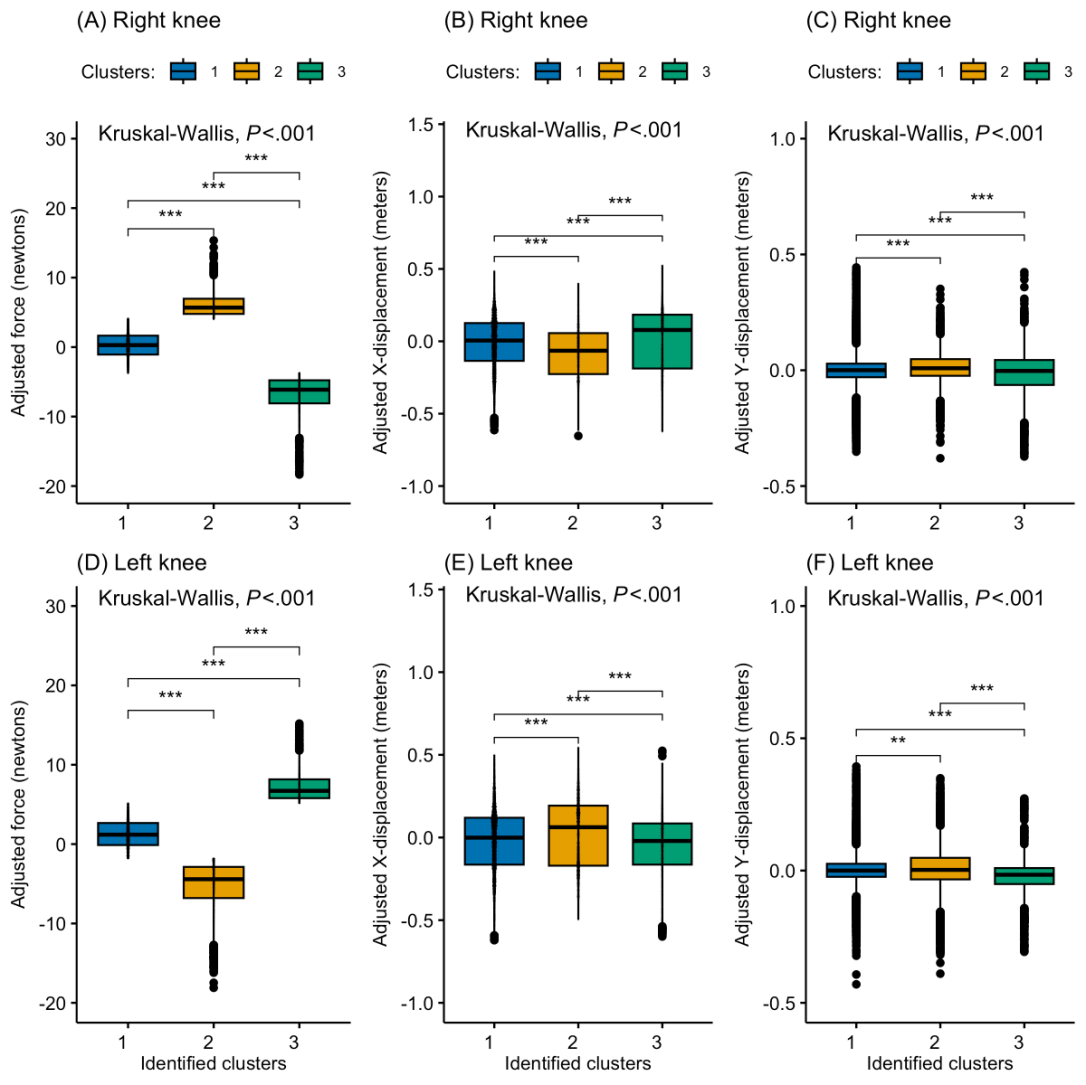
Comparative distribution plots of adjusted kinetic features (force and X-Y displacements) across the three k-means–identified clusters at the (A-C) right and (D-F) left knees. Statistical differences between clusters were assessed using Kruskal-Wallis tests followed by Dunn multiple comparison tests, with significance levels indicated (***P<.001; **P<.01; *P<.05). These results highlight variations in movement characteristics among identified rehabilitation patterns.

**Figure 7:**
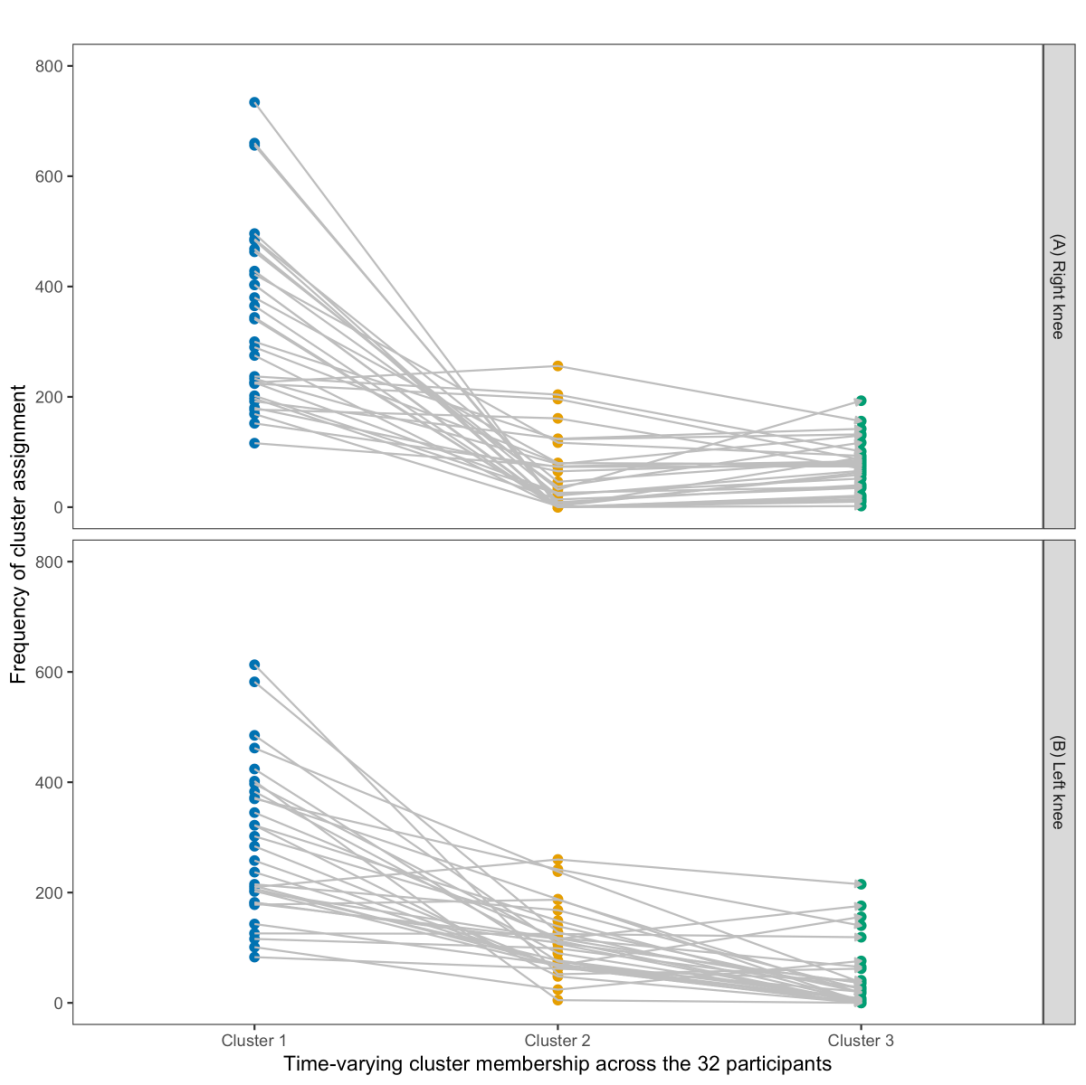
Slope graph visualizing temporal changes in cluster membership across the 32 study participants at the (A) right and (B) left knees. This plot captures how individuals transitioned between movement clusters over time due to the time-dependent structure of kinetic data collected from both knees, reflecting the dynamic nature of rehabilitation progress.

To assess whether significant differences existed in the distribution of multivariate kinetic features (force and X-Y displacements) across the three clusters for both knees, a multivariate Kruskal-Wallis (MKW) test [18] was used. This test was chosen due to the multivariate nonnormality of the bilateral knee datasets (Henze-Zirkler multivariate normality test: *E*=246.25, *P*<.001 for the right-knee datasets; *E*=219.34, *P*<.001 for left-knee datasets). The results indicated that at least one of the clusters significantly differed in distribution for both the right (MKW: *W*_6_=9204.35; *P*<.001) and left knees (MKW: *W*_6_=9973.54; *P*<.001). Subsequent univariate Kruskal-Wallis tests were performed for each kinetic feature at both knees, coupled with Dunn multiple comparison tests (Figure 6). There were significant differences in the distribution of the kinetic features between the three clusters at both knees, suggesting the identified knee-specific clusters were uniquely distinct. Figure 7 shows the frequency of times cluster membership varied temporally across the 32 study participants. The proportion of times participants were assigned to cluster 1, cluster 2, and cluster 3 at the right knee was 72.7%, 12.3%, and 15%, respectively. For the left knee, the proportion of times participants were assigned to cluster 1, cluster 2, and cluster 3 were 65.1%, 25.4%, and 9.5%, respectively.

There was a reverse observation of the relative positions of clusters 2 and 3 in the right and left knees of the study participants (Figure 5). Possible practical kinetic interpretations of the clustered data with respect to force measurements across X-Y displacements, given this reverse kinetic pattern in right and left knees, could be understood as follows.

#### Cluster 1 (Middle Position)

This cluster suggests intermediate force levels and displacements. In both knees, movements in this cluster might involve moderate force exertion and a moderate range of motion in the X-Y plane. These could correspond to exercises aimed at strengthening muscles and improving joint mobility without excessive stress.

#### Cluster 2 (Bottom Position—Left Knee; Top Position—Right Knee)

This cluster likely represents movements with lower force exertion and smaller displacements in both X and Y directions. In the left knee, these movements may represent exercises performed with less force and a limited range of motion. It could represent muscle weakening or joint pathology. In the right knee, however, these movements may involve higher force exertion but still within a constrained range of motion. The lower range of motion could represent joint stiffness due to arthritis. This cluster may represent the least physically fit group of individuals. In the right knee, it could represent moderate-intensity exercises, while in the left knee, it might involve active movements with moderate intensity.

#### Cluster 3 (Top Position—Left Knee; Bottom Position—Right Knee)

This cluster indicates higher force levels and larger displacements. In the left knee, movements in this cluster likely involve high-force exertion combined with extensive movements in the X-Y plane. These could correspond to activities requiring significant strength and flexibility, such as high-intensity exercises. The right knee, however, shows lower force exertion but still with extensive movements, suggesting exercises focused more on flexibility, agility, and possibly lower muscle activities. This cluster may represent the most physically fit group of individuals.

### Determinants of the Identified Clusters at Both Knees

The multinomial logistic regression analysis identified several significant demographic predictors of cluster membership for both right and left knee datasets (Table 1). For the right knee, being male was associated with significantly lower odds of belonging to cluster 2 compared to cluster 1 (OR 0.30, 95% CI 0.26-0.33) and to cluster 3 (OR 0.55, 95% CI 0.47-0.63). BMI also emerged as a significant predictor, with higher BMI decreasing the odds of being in cluster 2 (OR 0.95, 95% CI 0.94-0.96) but increasing the odds for cluster-3 membership (OR 1.05, 95% CI 1.03-1.06). Age was a significant predictor for cluster 3 (OR 1.02, 95% CI 1.01-1.03) but not for cluster 2 (*P*=.95). For the left knee, we found that all the demographic determinants of belonging to cluster 2 relative to cluster 1 were significant (.001≤*P*≤.008), whereas only BMI (*P*=.81) could not predict the likelihood of an individual belonging to cluster 3 compared to cluster 1. Gender emerged as the strongest determinant for the left knee data, with male participants being significantly more likely to belong to cluster 3 (OR 3.52, 95% CI 2.91-4.27; *P*<.001).

**Table 1:** Estimated OR^a^ and their 95% CIs for the demographic determinants of cluster membership, derived from a fitted multinomial regression model (N=32 participants)^b,c^.

Clusters and variables				OR (95% CI)		SE		*P* value
**Right knee^d^**								
	**Cluster 2 (reference: cluster 1)**							
		Male (reference: female)	0.30 (0.26-0.33)		0.061		<.001	
		BMI (kg/m^2^)	0.95 (0.94-0.96)		0.006		<.001	
		Age	0.99 (0.98-1.01)		0.002		.95	
		Pain score	1.03 (0.99-1.06)		0.015		.05	
	**Cluster 3 (reference: cluster 1)**							
		Male (reference: female)	0.55 (0.47-0.63)		0.077		<.001	
		BMI (kg/m^2^)	1.05 (1.03-1.06)		0.007		<.001	
		Age	1.02 (1.01-1.03)		0.002		<.001	
		Pain score	1.03 (0.99-1.06)		0.016		.11	
**Left knee^e^**								
	**Cluster 2 (reference: cluster 1)**							
		Male (reference: female)	0.63 (0.55-0.72)		0.069		<.001	
		BMI (kg/m^2^)	1.10 (1.09-1.12)		0.007		<.001	
		Age	1.04 (1.03-1.05)		0.002		<.001	
		Pain score	0.95 (0.91-0.99)		0.020		.008	
	**Cluster 3 (reference: cluster 1)**							
		Male (reference: female)	3.52 (2.91-4.27)		0.100		<.001	
		BMI (kg/m^2^)	1.01 (0.98-1.02)		0.009		.81	
		Age	0.98 (0.97-0.99)		0.002		<.001	
		Pain score	1.05 (1.01-1.09)		0.020		.01	

^a^OR: odds ratio.

^b^These results quantify the influence of demographic factors such as BMI, age, gender, and pain levels on the likelihood of belonging to specific movement clusters.

^c^Each of the multinomial logistic regression models for both knees’ data was adjusted for the primary kinetic features (force and X-Y displacements) and temporal effect.

^d^For right knee measurement data: percentage accuracy=77.82% (95% CI 76.29%-79.3%); area under the receiver operating characteristic curve=0.778.

^e^For the left knee measurement data: percentage accuracy=76.24% (95% CI 74.62%-77.81%); area under the receiver operating characteristic curve=0.762.

## Discussion

### Results Key Insights Enabled by the Slider Device for Home-Based Rehabilitation Monitoring

This study explored further an innovative method for knee rehabilitation using the Slider device, a novel tool that enables the remote acquisition of multidimensional kinetic data, including force and spatial coordinates, within a home setting. By facilitating objective real-time monitoring of rehabilitation progress, Slider represents a significant advancement in personalized physiotherapy. A previous study has already validated its feasibility and safety for empowering patients to perform presurgery physiotherapy exercises independently, eliminating the need for a clinical environment [3]. However, no study has yet examined and analyzed the multidimensional bilateral kinetic and kinematic datasets generated by users of this new device. This analysis aimed to uncover potential patterns that could transform raw data into actionable insights, enabling therapists to deliver data-driven, patient-specific interventions that may optimize rehabilitation outcomes. This study used and compared four unsupervised clustering methods to find three unique time-varying movement patterns or clusters based on the observed time-dependent, multidimensional datasets from the right and left knees of the study participants.

The application of multiple clustering methods provides a comparative perspective on clustering performance, reinforcing the robustness of the findings. We have identified these movement patterns outside of a clinical setting for the first time. Significant differences in the distributions of force and displacement between clusters show how differently participants move. Demographic factors like gender, BMI, age, and pain score also predicted membership in a cluster. These findings provide significant potential for individualized rehabilitation programs that enhance therapist accuracy and results. By leveraging ML and kinetic data collection, this study further demonstrates how at-home rehabilitation can be made more autonomous, reducing patient dependency on in-clinic physiotherapy sessions. The ability to remotely track movement progress offers a scalable solution for long-term rehabilitation, minimizing the need for frequent clinical visits.

Consequently, this study has effectively addressed the five open research questions by examining multidimensional kinetic data obtained during home-based physiotherapy. Using four clustering algorithms, the study found three different, time-varying, kinetic movement patterns for both knees. These patterns may represent different levels of fitness and recovery, answering the first question about how to find movement patterns in multidimensional kinetic data. The study answered the second question by showing that real-time clustering of movement data could help physiotherapists better understand how to meet the needs of each patient by showing how the clusters changed in response to force and displacement data. To answer the third question, the study confirmed that clustering methods, especially hierarchical clustering for the right knee and CLARA for the left knee, could tell the difference between different levels of movement efficiency and recovery states.

The mathematical modeling revealed that over time, participants progressed from erratic to more coordinated movement patterns, reflecting the evolution of the natural rhythm of motion during exercise, thus answering the fourth question on how movement patterns evolve. The insights gained from movement clustering suggest potential future advancements in physiotherapy technology, particularly in enhancing real-time feedback mechanisms of rehabilitation devices. These findings could contribute to the development of more adaptive and intelligent physiotherapy tools, improving their application in both research and clinical settings. Finally, the Slider device provided reliable, multidimensional kinetic data, enabling accurate cluster identification and analysis, thereby confirming its potential for remotely monitoring physiotherapy outcomes and answering the fifth question.

### Practical Implications of the Study

#### Identification of Movement Patterns

Clusters that differentiate based on movement patterns (such as the range and direction of motion) can help clinicians identify abnormal movement behaviors in participants. This is particularly useful in postoperative rehabilitation or in monitoring the progress of participants with musculoskeletal disorders. For a theoretical application in a clinical setting, cluster characterized by limited range and lower force output might indicate participants who are either in the early stages of recovery from injury or surgery or who are experiencing complications or poor recovery. Hitherto, such kinetic evaluations have only been possible if participants attended sessions in a gait laboratory or clinic [5]. Slider allows these evaluations to be done in a participant’s home using data from exercises done by the participants over the entire duration of their rehabilitation program. A possible weakness of this study would be that it is intended to account for movement of the knee but does not account for limitations in movement due to the flexibility of the ankle or hip. Such variables could have impacted the data collected and changed which cluster the participant fell into, however, no participants that participated reported having such issues before conducting the exercises, so such variables should not have been considered significant during data collection.

#### Tailored Rehabilitation Programs

Understanding which cluster a participant’s movement data fall into allows clinicians to tailor rehabilitation programs more effectively. By customizing programs based on the typical movement characteristics of each cluster, clinicians can optimize recovery times and outcomes. By understanding which cluster a participant’s movement data fall into, therapists can customize exercises that target specific movement deficiencies [6]. For a practical application, participants in a cluster showing high force and extreme movement ranges might benefit from a different set of exercises compared to those in a cluster with conservative movement patterns, possibly due to differences in pain tolerance, injury severity, or recovery stage. Remote data collection can enable clinicians to monitor progress in real time, offering greater flexibility in treatment planning and providing objective data for faster, more efficient feedback. This benefits both participants and care providers.

#### Prediction of Recovery Trajectories

The data clusters could help predict different recovery trajectories by identifying which participants will likely recover faster based on similarities in movement patterns. Participants showing gradual movement range and force improvements over time by progressing through clusters may indicate a positive recovery trajectory. Clusters may serve as predictive markers for rehabilitation outcomes, allowing for proactive adjustments in treatment plans [19].

#### Customization of Assistive Devices

Insights from clustering can inform the customization of assistive devices to better support individual participant needs based on the commonalities in movement patterns, unique movement limitations, or capabilities observed within each cluster. Participants in a cluster that shows significantly altered gait patterns might benefit from specific types of orthotic supports designed to compensate for those specific deviations [20].

#### Enhanced Understanding of Pathophysiology

Exploring cluster formation further can improve understanding of the pathophysiology of conditions, aiding diagnosis and treatment. Differentiating clusters by movement patterns can help identify causes of pain or dysfunction, such as muscular versus joint issues, and analyzing force and movement data can reveal significant correlations. This is significant as it allows therapists to obtain data regarding multiple variables at once during one exercise instead of having to test the functionality of the knee with the participant in the same room using multiple tests to achieve a similar outcome [21].

### Limitations of the Study

This study has some limitations that must be acknowledged from both statistical and clinical viewpoints. From a statistical perspective, the sample size of 32 participants, although adequate for preliminary temporal exploratory analysis, is not sufficient for generalizing the findings to larger and more diverse groups. A larger cohort would provide a more robust assessment of the observed kinetic patterns and enhance the reliability of the used clustering techniques. Furthermore, whereas unsupervised clustering effectively identified distinct movement patterns, the study design was deficient in longitudinal follow-up to assess the direct correlation between these patterns and long-term rehabilitation outcomes. Clinically, while the Slider device offers an option for home-based rehabilitation, the absence of real-time clinician supervision may lead to variability in movement execution and data quality, thereby impacting cluster accuracy. The predictive efficiency of the identified clusters in guiding treatment strategies remains uncertain without rigorous validation through randomized controlled trials or extended clinical monitoring.

### Conclusions

Three distinct, kinetic, time-varying clusters were identified for both the right and left knees, representing unique movement patterns, based on measurement data from the Slider exercise device. Data clustering performance was found to be knee specific, with hierarchical and CLARA clustering methods producing better results at the right and left knees, respectively. The kinetic patterns at the right and left knees were significantly different. Gender, BMI, age, and pain score were identified as significant demographic predictors of cluster membership, with varying impacts on cluster assignment for the right and left knees. Future research should also explore other clustering algorithms, such as density-based clustering (DBSCAN) and Gaussian mixture models. Additionally, the impact of various preprocessing techniques should be investigated, especially for time-dependent and cyclical data. Techniques such as Fourier transforms, wavelet transforms, and PCA for dimensionality reduction may also enhance the analysis of movement patterns.

Furthermore, future studies should develop additional kinetic features, such as velocity, acceleration, and jerk, to provide a more comprehensive understanding of movement patterns and improve clustering accuracy. Expanding this study to include a more diverse participant population would help assess the generalizability of the findings, refine personalized rehabilitation protocols, and address the limitations of this study. Finally, further longitudinal and trial studies could be conducted to correlate identified clusters with clinical outcomes, such as recovery trajectories and therapy efficacy, to validate the practical significance of the clustering results. Further case studies are currently being planned, which will expand the scope of data collected and study participants. It is expected that with further data collection, it will be easier to identify more accurately what subgroup knee replacement participants fall into, as well as what range of movement and force recorded can be considered optimal based on certain demographics. This method appears to be effective at remotely measuring movement and force for knee joint movement. Future projections for Slider device development will include artificial intelligence–assisted projections and therapy progress analysis to streamline the process for physicians.

## Data Availability

The datasets generated or analyzed during this study are not publicly available due to requirements of intellectual property protection but are available from the corresponding author upon reasonable request. The well-documented R scripts used for data analysis are publicly available for reproducibility of results [22].

## Abbreviations

CLARA: Clustering Large Applications
ML: machine learning
MKW: multivariate Kruskal-Wallis
OR: odds ratio
PAM: partition around medoids
PCA: principal component analysis
